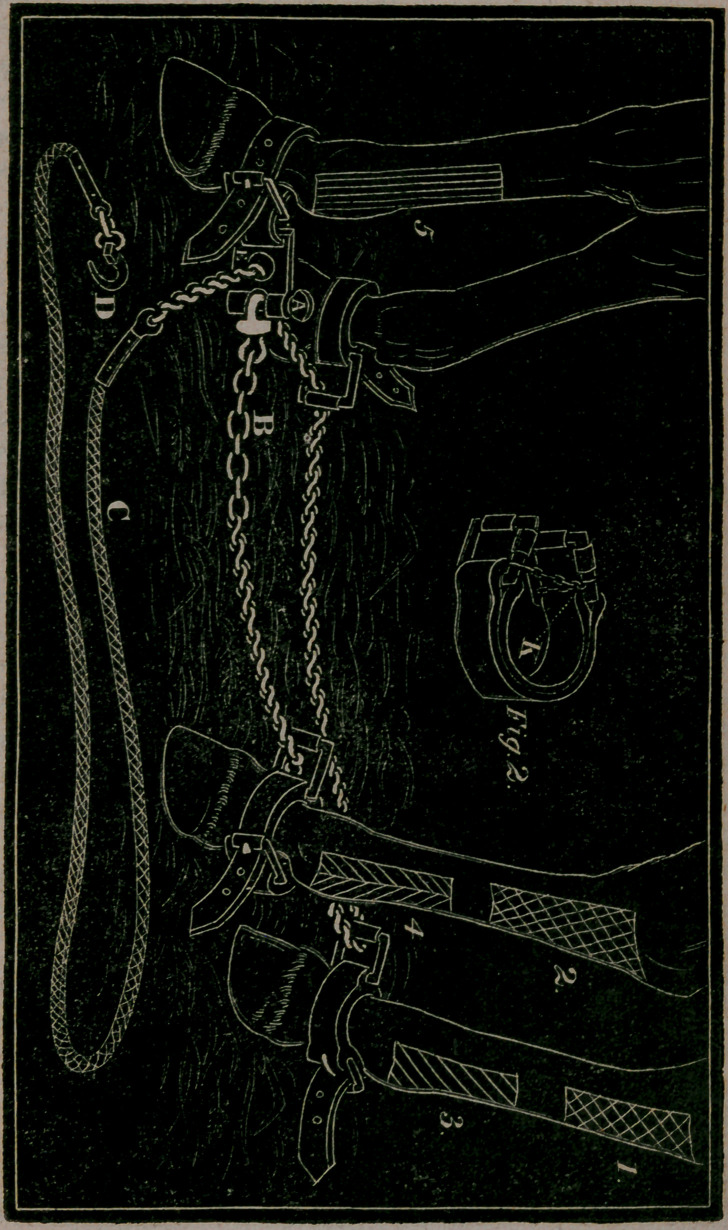# Of the Surgical Operations and Various Restraints It Is Sometimes Necessary to Place the Horse under for Their Due Performance

**Published:** 1818

**Authors:** James Carver


OF THE

### SURGICAL OPERATIONS,

AND

VARIOUS RESTRAINTS IT IS SOMETIMES NECESSARY TO PLACE THE HORSE UNDER FOR THEIR DUE PERFORMANCE;

SUCH AS,

### Castration, Nicking, Pricking, Docking, Blistering, Firing, &c. &c.

WHEN it is necessary to perform any painful or unpleasant operation on so powerful an animal as the horse, it becomes of consequence to secure both him and ourselves from the violent effects of his resistance, by subjecting him to restraint equal to the occasion. Horses are very unequal in temper, and bear pain very indifferently, though some I have again seen that would stand and let you run a red-hot iron through them even without flinching. It is, however, always prudent to prepare for the worst, and no important operation should ever be attempted without this precaution of casting; but which some idle smiths and farriers avoid, and call it a pack of d——d nonsense. Life is lost in a moment from the kick of a horse. A limb is fractured or broken in the twinkling of an eye. But how many painful days and nights, and even months, must not a person suffer before he is again able to walk. The smiths almost invariably do every thing by the side line and the *broomstick*.

To give directions on such occasions as these to the veterinarian is quite unnecessary; but as this work is intended as a guide to the inexperienced or junior practitioner, and those willing to learn, I shall not, I hope, prove *uninteresting or uninstructive* when I detail those minor matters. Humanity should be the fundamental principle by which we are to act on these occasions, and to subject the poor animal to no more pain and torture than is absolutely necessary. The resistance of the horse is terrible, while that of the huge elephant is silent murmuring, with groans and tears that would almost pierce the heart of a rock. This I have many times been an eyewitness to during my long residence in India.

The lesser restraints are various: among them may be noticed the *Twitch* and *Barnacles*, as so called. The Twitch is a very necessary and useful instrument in a stable, though often unnecessarily used, which always has the ill effect of rendering many horses vicious to resist its too frequent application. There is no part more tender and more susceptible of feeling than the nose of most animals. By the nose you may drive or guide the most unruly horse, almost with a twine. By the nose man and dog can pin and subdue the strength of the strongest and most ungovernable bull. In many instances blindfolding will do more than the Twitch. By a firm, but soothing manner and some coaxing, with a little *sugar* or *salt*, but *no flogging*, you may not only do and perform wonders, *but subdue the most violent horse in the world.* Horses can use their fore legs as well as their hind ones, and persons should be on their guard in blindfolding a horse, for he will not only strike out, but even take aim in doing so. Barnacles are a sort of clams used by the smiths as they use a Twitch, and are admissible only when assistance cannot be obtained. When one of the fore feet or legs wants a minute examination, it is always prudent to have the opposite one held up; if the foot is then elevated to kick, there is always sufficient warning for the operator to avoid the stroke, and without the use of this precaution the practitioner runs some risk, which he may always avoid.

### THE TREVIS.

This machine is the very utmost limit of restraint, and is very seldom used but by smiths to shoe very powerful and unruly horses. Whenever it is necessary to have recourse to this machine, great care should be taken to bed and bolster all the parts of it that are likely to come in contact with the horse's body. In France this machine is used to much greater advantage among their smiths; and during my residence in that country I have seen horses very dexterously shod. Their smiths shoe very different from ours; and when they come to this country we are both astonished and surprised to see how one man will handle a horse by himself, while here they say it takes three and four men to shoe one. The Germans shoe on a block; and the French have a round cross bar in their Trevis or across their stalls in the shops, and the hind foot is taken up and fastened there by the fetlock joint, by straps and cords, and then they cut and slash away with their buttems. I have, however, seen many horses ruined and destroyed by the Trevis; at least their aversion to restraint has been such that they have died under their own resistance: it should, therefore, never be used but by patient dexterous men. As for my own Trevis, having always taken the greatest precautions in using the machine in the way I have made it, I never had an instance where it was even offensive, or that I could not put it on a horse the next day if necessary. The sort of Trevis I mean, is like unto that in Baldwin and Thomas' stables in Fourth street; and if properly made on that principle, with a proper screw, canvas girt, and broad leather (not rope) back strap, it is in the hands of a prudent man one of the best machines I know of for Nicking, Docking, and Pricking. If, however, I must give my real and honest sentiments respecting these things (which are only invented by the ignorant to gratify the pride of man) they are all entirely useless, excepting docking, and it is only a cruel and barbarous custom. Docking, however, is so simple, and, if properly done, is attended with so little pain or danger, that I do not object to it; it might always be done, and at the same time combine both nicking and pricking, without resorting to these butcher-like operations. And I will maintain that if docking is properly executed on my principles, that a horse with ever so bad a tail may be made to carry well, without resorting to either of the two former operations, viz. nicking or pricking. This operation I have performed in two ways during my stay in India, and with always as good an effect as by having recourse to that barbarous custom.

### THE SIDE LINE.

The side line, such as used at the Veterinary College of London, is a good thing, and may very conveniently be used in some of the minor operations, particularly on such horses as are liable to strike behind. It consists simply in placing a plain hobble strap on one hind leg, and then passing the end of the rope attached to the withers, bringing it back again under the neck, and over the other portion over the neck, so as to leave a slipping collar over the neck as it were, by which the hinder leg should be drawn forward as far as it can without elevating it from the ground. By this displacement of one leg, the animal is perfectly and effectually secured from kicking; it is, therefore, a very useful means of restraint in slight cases. I would not, however, recommend the risk of any important operation with a side line only.

### ON CASTRATION.

Castration is the operation by which an animal is deprived of his testicles, so as to incapacitate him for propagating his species. When performed on the male, it is called gelding; on the female, splaying; and the latter is performed in India on mares as common as on sows in Europe. A good deal of difference of opinion exists as to the proper time of castrating colts. Bulls are often castrated at eighteen months or two years old; but it is observable that they grow a good deal larger, and fatten sooner, when it is done at ten or twelve days after birth: and it has by experiment lately in England been proved, that they not only become much fatter, but less ferocious and more active. In colts it is sometimes performed at three months; at others it is deferred till twelve months: but when performed so early there is a difficulty attending it in the horse, which wants explanation. Mr. Coleman observes that the testicles of the fetus in the human subject, in horses, asses, and most other quadrupeds, is situated below the kidnies in the loins, and they remain in this situation in birds during life, and sometimes in man and horses. But as the testicles do not come down into the scrotum, but remain in the abdomen or belly, there is some difficulty in castrating colts at twelve months old. The spermatic vessels which must be divided are of course very short, but they begin to grow longer exactly as they descend, and it is not until some time after their descent that they begin to secrete: in the human subject not until the thirteenth or fourteenth year; but they perform this office at ten, eleven, and twelve months in the horse, and they are the only glands that possess this peculiarity, all the rest beginning to secrete their proper fluids soon after birth, as the liver, kidnies, &c. &c. It is a curiosity how the testicles of the horse descend into the scrotum, not by gravity, as was supposed by Dr. Hunter, it being possessed of a muscular power which enables it to draw the testicles down when they come to the orifice of the abdominal ring; they of course meet with the peritoneum, and carry part of it with them much the same as the sack in hernia.

### *College Mode of Castration.*

We first begin by dividing the scrotum or bag in which the testicles hang, and then the tunica vaginalis, or second coat that covers it; after this the test slips out of the bag or tunic. We then divide every thing with the knife, but the vein and artery. We divide the vas deferens higher up than we do the blood vessels, and with it remove the epidermis or straight cords. We then get a pair of clams, or tournaquet as you please to call it, and rolling some tow round it, embrace between them the blood vessels *near the body of the testicles*, so that if bleeding or hæmorrhage should take place afterwards, we may be able to get at them. Then take the actual cautery or firing iron, (not too hot; if white, reduce it to a red heat by rubbing it on a smooth piece of plank or old shoe) and scrape the vessels with it, so that we may leave the extremities *ragged*. The common farriers do not take this most necessary precaution, which is the only particular in which we differ from them in performing the operation.

After dividing the vessels in this way, we then sear the ends, and by sprinkling a little rosin on the part, it will keep them plugged up, and the more readily stop them from bleeding. The pressure made by the clams should be very considerable to stop the circulation, but at the same time be careful not to bruise them *by too severe pressure*, or you bring on gangrene and mortification; this, therefore, requires judgment. We should not remove the clams until we are certain that we have plugged up all the vessels, and this may be tried by gradually opening the clams, till we see whether the blood comes through their extremities. If this should be the case, we again employ the necessary pressure, and make use of the hot iron and rosin till it does stop. It is a very common and received opinion of smiths and farriers, who, unacquainted with the structure, think that by removing more or less of the epididymus,* gives more or less courage to the animal; but this is a very mistaken notion, because it is not the duct, but the gland that gives courage.

After the operation is performed, we should strictly adhere to the antiphlogistic plan for two or three days. You should tie his head up, give no food for twenty-four hours, and bleed some hours after the operation to the quantity of three or four quarts, provided no hæmorrhage has taken place. If the swelling gets high, give walking exercise to promote absorption. You may also give purgatives and diuretics after the third day, and when suppuration comes on, we must pursue an opposite plan, and give mashes made of good meal or shorts or other nutritive food; and if the inflammation should continue, you must insert rowels. I have dressed the parts after the operation with spirits or tinctures; but during my latter

* The serpentine, convoluted, or curled artery, is the spermatic; the straight one is the cord or epidermus. It should also be recollected, that no organ in the animal body demonstrates so powerfully its sympathy with other remote parts; and for this reason I gave the above caution respecting mortification.

A case of this kind occurred during my practice on Long Island, and it was with some difficulty I succeeded in recovering the horse. For want of this very precaution many colts are killed, and no one knows the reason why.

practice, I have applied only dry lint within the edges of the divided scrotum. In colts no dressing is required; but in the old horse, who gets stiff, it may sometimes be necessary. In this operation, the only precautions that are necessary, is to give the due degree of force to the clams, and to be careful in searing: it is better to sear too little than too much; if too much cauterized, inflammation is sometimes apt to make its way up the cord, which must be guarded against. Under all circumstances, if the horse can be turned out to graze during the day, nothing will tend more to facilitate the cure.

### DOCKING.

THIS operation consists in the removal of a portion of the tail, now conveniently practised by what is called a docking machine, which takes off at one stroke the part determined on, it having been previously freed from hair. In colts the searing should be very slight, and in the adult horse it never should be harsh, for even if altogether omitted, the hæmorrhage would never be great or serious. In very full and fat horses, I have frequently applied (as we do at the College) a little powdered alum. The most serious consequence in docking is, that, slight as the operation appears, mortification will in some cases take place, and lock-jaw in others. One thing, however, should be particularly attended to, which is, not to use the horses before they are perfectly well. The neglect of this precaution is often attended with serious consequences, and too frequently brings on lock-jaw.* In such a case, I would always recommend a second removal of another portion of the tail, and to sear it also; but in mortification terebinthinated dressings only must be applied.

### OF NICKING.

Respecting Nicking, there are various methods of performing this most cruel, barbarous and *butcher-like operation;* and I most sincerely hope that every man in possession of his rational faculties, and possessed also of common humanity, will never consent to have this operation performed. Pricking, a less painful operation, is now so much in use by every stableman, that nothing further on the subject, I trust, need be said.

### ON CROPPING.

Thank God! custom has also nearly abolished this useless, shameful practice. Circumstances may however occur, though very seldom, to render it necessary. After all, if we reason rightly, though we do possess a horse, with flapped

* A case of this kind lately occurred at Messrs. Thomas & Baldwin's. It was perhaps an oversight, for I know of no two men who use more caution, or that pay more strict attention to the animals under their care than they do. There is, however, a mode of performing this operation to answer all the purposes of nicking and docking combined.

ears, if he perform his journies and labours well, it is only foolish pride that can induce us to do it. One thing is evident, it makes horses very shy about the head; to lessen which, if it should be done, a bridle and halter also should be used without a fore part or fronting, till the ears are well; the bridle should also be made to unbuckle at the side of the bit, so that the head-stall may be dropped on, without the hand being raised to pass it over the ears. This necessary precaution may materially operate in lessening the shyness, which otherwise long remains, and which is never wholly lost if force and cruelty are afterwards used.

### ON BLEEDING,

### OR VENESECTION.

**Bleeding** is a well known operation, by which a due quantity of blood is taken from an animal with a view to cure some existing disease, or it is performed in disease as a mode of cure. When blood is taken from an artery it is usual to divide it, as by this means the artery contracts and hæmorrhage is spontaneously stopped. When bleeding is performed from a vein, a longitudinal opening is made by means of a fleam struck with a stick, or a fleam pushed forward by means of a spring, which is in use for a large rolling vein. Blood is also sometimes drawn by means of cupping, and by the application of leeches to any part. Blood is also sometimes taken from the palate, from the jugular, the plate vein, and the superficial veins of the thigh. Local bleeding is also sometimes performed in the metacarpal or canon vein, running down on the posterior or back part of the pastern, for all inflammations of the feet, but more particularly in founder. Bleeding in this part should always have the preference, as a quart of blood taken from it in all affections of the feet, will do more good than three times that quantity from the general system. This is a nice operation, and should be done with some caution; for as the artery, which in this place is very large, lays immediately in contact with the vein anteriorly, there is great danger of wounding it, unless performed with judgment.

So much am I an advocate for bleeding in this part for affections of the feet, that I think it well worth while for a person to study the structure of this part well, in order to perform this operation nicely. In running the finger down anteriorly along the pastern to the heels or cartilages of the foot, it may be found and felt, and by keeping the finger of the left hand on the vein to raise it, and placing the spring lancet on the vein below, the operation is easily performed. Sometimes it bleeds but slowly, at others very profusely, but never so as to create danger, provided your fleam or lancet is not too large. The common sized human spring lancet is the best, though it may be performed with a small sized fleam, provided you do not strike too heavy a stroke. It may always be stopped by keeping your finger a few moments on the orifice, or by binding a piece of tow or a hay-band round it.

In bleeding from the jugular, some precautions are necessary; but a ligature is entirely useless, as the vein may always be found by a little exercise, and by pressing against it with the middle finger of your left hand, and keeping it there a short time, the vein will become sufficiently prominent and visible to direct you where to strike. The accompanying plate will, however, be your best guide.

Never let the blood fall on a dung-hill, as the quantity taken by this means cannot be accurately ascertained, and the amount drawn must be determined by the nature of the disease, size, age and strength of the animal. Every stable-boy will tell you he can bleed; but can he tell you the state of the blood when drawn? or can he tell you why the difference of blood while in the body and when out of it; and how this change is produced? how long it should be in coagulating, and the nature of the disease from its cause? Can he tell or explain to you the difference of arterial and veinous blood? and whether, when performing the operation, he has opened by mistake a vein or an artery?—and if so, how to stop the one, and how to prevent the animal from dying by hæmorrhage? These, and many other things respecting horses, show the absurdity of people's talking about matters they have only got by rote from Tom, Dick and Harry. Many people, and very many of them, who profess to know a great deal of their diseases, their causes, their symptoms, &c. without rhyme or reason, bleed in the mouth, or in the tail—cut out the lampas, the hooks, and practise many such absurdities, and cannot give a rational reason why or wherefore.

In stopping the orifice when you bleed in the jugular, never pull the skin (as many do) from the vein underneath, as it is apt to produce a flow of blood in the cellular membrane, by which very serious cases often happen. Not less than twenty horses have I seen destroyed during my residence at the college, by this operation being done in a bungling manner by the smith or common farrier.

In drawing blood from the veins of the extremities, as before mentioned, for local inflammation of the feet, the bleeding stick should be avoided and the spring lancet only should be used. The most proper place for general bleeding, is two inches below the division of the jugular vein, where it branches off, as described in the plate.

Practitioners are apt to be too indifferent as to the state of their instruments. Lancets and fleams should always be sharp, and very highly polished.

The temporal artery may be opened in cases of staggers. This vessel can be readily detected at three or four inches below the root of the ear, in a line towards the nostrils, and may be punctured with a common lancet, in the same manner as a vein, and afterwards secured in the same way. But when opened for furious delirium, there is no necessity for securing it; the collapsion of the artery will take place soon enough, and often so soon as to require the opening of the jugular. Bleeding in the mouth is seldom resorted to by the regular bred veterinarian, as no good but under one circumstance can possibly be derived from it. It is, however, the sheet anchor of every common vulcan of this country; for let what will be the matter with their horse, bleed him in the mouth is the common cry; and little do they know the danger of even attempting to bleed in this part; for it should be understood, that between the fourth and fifth bar of the superior maxillary or upper jaw, there is an opening or hole in the bone, through which the palatine vein and artery runs. These are very large, and when wounded, very great hæmorrhage is sure to follow, and is often attended with very serious consequences from the great difficulty of stopping it, which only can be done by one way, and that is the actual cautery. Under no circumstances but one would I ever be induced to open this part, and that is when a horse is labouring under very strong delirium and madness in staggers; for I know of no disease that requires more profuse bleeding: and to show the violence and danger with which it is attended, I will relate the following case that came under my eye during my residence in India.

A very fine horse, the property of sir Charles Blunt, was attacked with this complaint, and when I first saw him he was labouring under such a high state of madness that twenty men were unequal to the task of holding him. Under these circumstances, as he was literally knocking himself to pieces, I availed myself of a favourable moment, and pulling the tongue from the mouth, I divided the vein and artery; the hæmorrhage was very profuse, and the horse bled till he dropped for dead. I then seared the part with rosin and aloes, and left him in this weak state for several hours, when he got up. By proper attention, use of medicine and other necessary aid, the horse recovered.

In another case similar to the above, I opened the jugular on both sides of the neck till syncope took place; and the other necessary aid being applied, the horse also recovered.

In bleeding in general, the quantity drawn should always be measured; this may be easily accomplished by receiving the blood in a proper graduated pint or quart tin measure. In a very high and active state of inflammation, such as Staggers, inflamed Lungs, Founder, &c. never less than five, six, seven, eight, and sometimes even ten quarts of blood, should be taken away; but when extensive and profuse bleeding is necessary, it should be done by those who know how to judge of the state of the horse, heaving of the flanks, &c. &c.

In all important inflammations it is of consequence to draw the blood from a large orifice, and that as quickly as may be; the increased action of the vascular system appears to be more readily checked by a sudden evacuation of blood, probably by a sympathetic effort by which the vessels recover their tone by a hasty depletion.

It is necessary to remark here, that all blood, when first drawn, presents indications that should not only be attended to but studied. When blood is taken from an healthy subject, and suffered to settle or coagulate without disturbance, it soon separates into two portions; one is fluid and yellow, called the serum; the other forms itself into a jelly like substance, and swims in the serum: but when the subject is labouring under inflammation, or inflammatory disease, both these parts undergo alteration, and exhibit different appearances to what they do in health: in violent inflammation the quantity of serum is small, and not particularly altered in colour, though sometimes it is milky; but the cruor or crasamentum, which is the jelly like cake in the centre, instead of being easily broken, becomes tough, and instead of being of a bright scarlet, its surface is yellow for two, three, or more inches of its depth. Such blood is called sizy, and exhibits great marks of inflammation, and thereby becomes a very important monitor for us to repeat the operation so long as the same appearances last, provided the horse's strength will bear it. When, on the contrary, blood is drawn which exhibits much serum, with the coagulum red, but thin and without tenacity, we are warranted in considering it a case marked with debility, in which a repetition of bleeding would be dangerous.

Although a vein is not strictly a circumscribed cavity, it has no communication with the air, and when once exposed, if the parts after the operation do not unite by the first intention, the vein is liable to great mischief. The treatment under such circumstances will therefore appear from the following cases which occurred at the London Veterinary College.

A horse was admitted on the 30th of August which had been bled in the jugular vein on the near side six days before; the orifice of the wound was much inflamed and swelled to the size of an apple; it also discharged, and frequently bled. On further examination with a pròbe, the cavity of the vein was found open; the actual cautery was applied to the lips of the wound, which immediately prevented any further discharge of either blood or matter.

On the 1st of May—Suppuration took place from the same wound; the cautery was repeated; and again stopped as before.

2d.—No discharge from the vein, and the neck was ordered to be fomented with warm water.

3d & 4th.—No discharge; fomentation as before.

5th.—The wound discharged a small quantity of matter, and the cautery again applied. The next day the animal was sent for, and as the swelling and inflammation had abated, he was suffered to be taken from the College.

10th.—He was again returned to the College, and on inquiry it was found that by some accident the coagulum was torn from the surface of the cavity, and the discharge considerably increased; the inflammation and the swelling extended up the vein as high as the head The cautery was again applied, and the discharge stopped. The horse was bled from the opposite vein and a dose of physic given, which operated the next day.

15th.—The wound again suppurated, and the cautery was repeated to a greater depth.

16th & 17th.—No discharge; the tumour was blistered, and by the 18th much reduced.

19th.—The granulations protruded through the orifice, but no discharge. A small quantity of powdered vitriolated copper was sprinkled on the part.

21st.—A small orifice was observed in the centre of the wound from which matter discharged, and on introducing a probe the cavity was found superficial. A drachm of the same copper in solid form was introduced into the wound, and a large poultice applied over it.

22d.—The neck was fomented with warm water, and the poultice repeated.

23d.—The inflammation and swelling gradually abated, but the orifice sloughed and again suppurated; the cautery repeated as before.

24th.—A small discharge from the orifice, and the cautery again repeated.

26th.—The surface of the wound sloughed, and healthy granulations appeared; the wound was afterwards dressed with simple ointment, and the granulations sprinkled with blue vitriol.

Under this treatment the horse was discharged from the College on the 10th of June, radically cured.

### CASES AND DISEASES;

### UNDER WHAT CIRCUMSTANCES THEY REQUIRE BLEEDING.

The cases and diseases that require bleeding, are those under which active inflammation is going on, and its consequences; for instance, wounds and sprains of the muscular or tendinous parts, bruises, &c. It is also proper in the beginning of all disorders or cuticular eruptions of the skin; in large swellings of the body or legs, arising from a plethoric state; and in swellings of the legs and heels, which are attended with much inflammation. Bleeding is very often the speediest method of giving relief in the beginning of fevers, to which horses are very liable; it is also necessary in acute pain, as in gripes, colic, strangury, or suppression of urine; in rheumatic affections, where pain causes either lameness, stiffness or contraction of the muscles, which by farriers are commonly called the cords; in inflammation of the eyes particularly, the lungs, the liver, the bladder, the stomach, the intestines, and other viscera; in apoplexy, vertigo, or giddiness, &c. On the other hand, people in this country should be cautioned from bleeding so frequently on the road as they do in hot weather, and then only under urgent cases should we have recourse to it.

The *Bursæ Mucosæ*, are the bags called Wind-galls, &c. whose office is to emit a lubricating mucus, to facilitate the motion of the tendons of the animal body, when they play one upon another, or upon bone. Men not acquainted with the anatomy of parts, or the function of mucous capsules, open them to let out the fluid. The immediate effect of this absurdity, is the total disappearance of the tumour; and if the edges of the wound do not unite by the first intention, violent inflammation immediately takes place, and death is very often the consequence: the only mode of shutting or stopping them is as related in the before mentioned case, the *actual cautery*.

During my residence at the Veterinary College, Mr. William Sewell, the assistant professor, was called upon to visit a horse belonging to a gentleman, and on examination he found one of the mucous capsules between the flexor tendon or back sinew, and the long ligament that supports the two sesamoid bones opened by some accident that had happened two or three days before. The leg was considerably inflamed and enlarged above the knee joint, attended with a considerable discharge. The cautery was applied to the surface of the wound, and fomentation and poultices to the legs. A purgative was given, which operated well, and on the fourth day the discharge of synovia again appeared, but the cautery being again repeated, in less than twelve days the discharge stopped. The leg, however, continued to swell, and being inflamed, a liquid blister was applied from the fetlock to the bend of the knee, and diuretics given every night and morning. When the blister had ceased to discharge, to keep the parts moist and preserve the hair, a cold poultice of bran was ordered with a second dose of physic, which, by the assistance of another blister, completed the cure. The horse was discharged in one month perfectly cured and sound.

### OF WOUNDS IN THE VEINS,

### AND THE

### MORBID CONSEQUENCES OF BLOOD-LETTING.

When a large vein is divided, it should be secured by a ligature above and below, or the anastomosing branches will continue bleeding; but when the smaller veins are divided they stop spontaneously. A divided vein will again unite and become pervious, but an artery will not. There very frequently follows serious consequences from blood-letting, which are of two kinds: the one shows itself by simple effusion of blood from the punctured vein into the cellular substance, occasioning inflammation in the parts around; and another where the vein itself takes on inflammation (as in the before mentioned cases) from a puncture through the opposite coats, or perhaps from causes we are unacquainted with. In the first of these cases there occurs a *thrombus* or *ecchymosis*, (an effusion of blood under the skin) from an effusion of blood into the surrounding cellular substance, originating frequently from an improper mode of *merely closing* the cavity or orifice. In bleeding the operator generally draws the skin too much out in order to introduce the pin, by which means the blood becomes effused; or sometimes the openings between the skin and coats of the vein are not correspondent, which produce the same effect. Whenever this takes place, press the effused blood carefully with the fingers, and if the bleeding appears not likely to come on again, put no pin at all; and if one has been introduced, remove it and let the horse be watched, and his head tied up; and in all cases remove the pin in twenty-four hours, (better to watch the horse for two or three hours, than have a swelled neck, which may be attended with very serious effects, at all events a great deal of trouble) as that alone will frequently irritate and do mischief. If this effusion should occasion after inflammation, apply a cold solution of sal ammoniac and vinegar to the part, or a solution of sugar of lead. If the effused blood should afterwards fluctuate, or matter form, make a depending orifice to form a seton, or use the other remedies as before named in those cases. It is not uncommon under the before mentioned causes, that ulceration often proceeds and sinuses form; still the morbid action extends, and sometimes the parotid gland becomes swelled and tender; the whole neck also becomes stiff and painful, and the animal experiences a difficulty in eating and drinking; at other times the disease proceeds downwards, and inflames the whole course of the vein till it reaches the heart, which soon destroys the animal.

*Treatment.*

We should, under these circumstances, direct our efforts towards saving the vein. When the disease first appears, it may very often be stopped at once, by a single application of the budding iron (not too hot) to the outer edges of the wound.

It will, however, sometimes be necessary to apply this for two or three days, until the oozing or moisture ceases. If, however, the tumour increases, proceed as before directed.

### OF BROKEN KNEES.

Broken knees is a term sufficiently common among horsemen and farriers; and though the treatment is referrible to the direction of wounds in general, yet my readers will expect some direction for these cases, which so frequently happen.

Horses when they fall extend their knees forward to save their head; and as the fore parts usually descend with great violence, so there happens very commonly some laceration of the integuments or skin of the knees. When this extends into the cavity of the joint, which may be known by the extreme lameness and swelling of the parts, as well as by the flow of a particular slippery mucus, called *joint oil*, very different from the common matters that issue from wounds, the case must be treated exactly according to the rules so fully detailed under wounds or open cavities of the joints; but when there is only simple laceration of the skin of the knee, treat according to the extent of the injury. However, great or small, let me beg and advise that you carefully abstain from the heating applications of what farriers call *hot oils*, which are generally composed of such irritating materials, that a man might as well throw gunpowder or brimstone into the fire to put it out, as to apply any such remedy. Sufficient inflammation will ensue without such aids, or the torments of turpentine, ardent spirits, vitriol, &c.*

* It is literally true, that every book on farriery, written *before the establishment of the Veterinary College*, is erroneous as respects the treatment of wounds in the joints. No one, I presume, will suppose that any disease can be successfully treated, without a due combination of theory and practice, directed by skill and sound judgment; for they mutually support and correct each other. Without these two essential points, nothing but error will be the consequence. In order, therefore, to justify the opinion which I have so often advanced against the erroneous method of shoeing, as now so *bunglingly practised* by almost every smith in this city, and throughout the United States, let us now consider the practice of the common farrier in the case of open joints. We shall soon see that his science consists in the method of injecting into the cavity of the joint corrosive mixtures of all kinds, but more particularly a mixture of turpentine and oil of vitriol or corrosive sublimate, dissolved in lime water or tincture of myrrh. Now let the common sense of every man who has a slight knowledge of the nature of those *soft, bland, emollient articles*, determine, whether the devil must not have put those things in the farrier's head—for surely it must be nothing but the height of madness for a man to think of applying such hot, irritating substances to the joint of a living animal.

These compositions, however, are considered among the illiterate as a secret, and these men, under the assumed title of veterinary surgeons and professors of horse medicine, even offer them for sale at high prices; while on the contrary, every judicious observer will and must see, that any corrosive infusion put into the cavity of the joint, can have no other effect than to produce the highest state of inflammation and swelling, which increases the violence of the symptoms in spite of their *grand secret*. The consequence is, that the upper part of the limb wastes and decays, and the poor animal suffers under their *unpardonable ignorance* until the grim monster death closes the scene of misery.

A gay young buck of this city who assumes to a knowledge of these things, though perhaps ignorant of the quality of five medicines in the whole

If the wound be large and the laceration extensive, and you suspect sand, dirt, or any foreign substance to be within, wash it with warm water, and apply a saturnine poultice. But if no foreign substance is within, never wash the wound; the lymph of the blood being the best *natural balsam* you can possibly apply: the part, however, may be touched slightly with a little tincture of myrrh and aloes, which will be found sufficient. Turlington's balsam is also one of the best applications to wounds of this kind (where suppuration is not wanted) you can apply. But to those who have horses for sale, to prevent or lessen the blemish, is as much a consideration as to heal the wound itself. Three circumstances are therefore desirable: to bring the hair on, to bring it on smooth, and to lessen the scar. Nothing therefore tends so much to obtain these ends, as to avoid the common farriers' heating applications; and, in addition to this, so soon as the part is actually skinned over, materia medica, applied to me in a case of this kind. The actual cautery and fomentations, with the usual applications, were accordingly applied. The horse was in a great deal of pain, and literally stood on three legs, though he had then been in the infirmary a week. On coming one morning to the stall, the gentleman cried out, "Where is the doctor? (the inflammation having sufficiently abated to warrant the application of a blister it had accordingly been done,) doctor, what the devil have you been about? why, you have been blistering my horse?" Yes, sir. "Why, you are a madman—who the devil could dictate to you so absurd a thing?" Years of study, and the practice of some of the first medical men, warrant what I have done. "O that be d——d; you know nothing at all about it; and I shall take my horse away, if he is not well by to-morrow."

Let the cap fit where it applies. Boys must not presume to dictate to men of experience, who have spent thirty years of a life in acquiring a knowledge of these things.

apply a mild blister—nothing will produce hair sooner; it absorbs the edges of the scar, and by removing the old hair, it *stimulates to the reproduction of the new of one colour;* and this very simple application, and the way to apply it with proper effect, is what many a man would cheerfully give pounds sterling to know. Many recipes are given to produce new hair; but most of them are useless. Nothing acts specifically in this way; but whatever gently stimulates the skin, may assist generally. But remember, never apply the blister before you are thoroughly sensible it has cicatrized, or skinned over. Apply therefore the following:

Take Venice turpentine . . . . one drachm.
Goose grease . . . . . . . one ounce.
Mix with the white of an egg.

### OF OVERREACH.

This sometimes happens from weakness, but very often from bad shoeing, leaving the toes of the hind foot too long. It is usually a blow inflicted on the heel of the fore foot by the toe of the hind one. But when it strikes the flexor tendon or back sinew, it produces inflammation, which requires no variety in treatment. Remove the cause, and the effect ceases.

### STRAIN OF THE STIFLE JOINT.

The ligaments of the patella, or stifle joint, may be strained or inflamed by violence or blows. And as the former affection may arise from a variety of causes, it may be easily distinguished by a peculiar dragging of the limb; and this may always be known by the *circular direction* in which the leg is carried during motion, purposely to avoid bending the joint. The heat and tenderness will also serve to guide the practitioner in this instance.

Sometimes the muscles of the thigh, and not the joint, become extended, and produce the lameness; in which case, the tenderness will be found within the groin, and not around the patella or stifle. The treatment, however, is no wise different from the former, except that sometimes a rowel inside the thigh has benefited this, but is of no service in the other.

### OF THE PUMICED FOOT.

THIS is a very common affection of the foot, and is the very reverse of concave feet. It is also a very common effect of both acute and chronic founder; an account of it very properly following the affection called Founder.

Pumiced feet is in many instances the effect of inflammation both of the sensible and horny laminæ, and the vascular increase may be an *acute* or *chronic* one. When they are the effect of the former, the complaint immediately follows an attack of acute founder, and is brought about by the very same causes; but when produced by a slow chronic inflammation, its attack is much more insidious and slow, and its approaches gradual; the front of the foot is observed to fall in, and the sole to become nearly flat, at which time the animal begins to falter, and is sometimes very lame, at others can move tolerably well. Mr. Coleman says, that the large wide feet of horses bred in marshy, low, wet countries, are the most subject to it; and such feet are but ill calculated to stand the battering of pavements or hard stony roads. Nor is the inflammation producing this evil altogether the same with that which contracts the foot; for in most cases the former is attended with an over increase of horn, whereas the latter is attended with a decrease of horn, and both the walls, as well as the sole, become thinner. The sensible and horny laminæ, as before observed by Mr. Coleman, lose their elasticity, and do not secrete horn; but a considerable quantity of diseased substance, which, with the weakened structure of these supports, displaces the coffin bone, drawing the crust with it, and greatly increases the natural obliquity of the hoof, and the pressure that the coffin bone, thus displaced, makes on the fleshy sole, occasions an absorption of its own. The sole, therefore, unable to support or resist the superincumbent weight, loses its concavity, and thereby yielding to the altered parts above it, bulges into convexity, and becomes deranged in structure as well as situation.

### *Treatment.*

The treatment of such feet can only be palliative, as a removal of the deformity has never taken place. Blistering the coronet will, in the incipient stage of this disease, stimulate the foot to an increased secretion of horn. Mr. Coleman uniformly advised pressure on the sole, by causing the animal to stand on a flat marble slab placed in front of the stall, and the feet placed in pitch. This is condemned by some, and is wholly avoided by others as being too painful, and thought to aggravate the disease. Pumiced feet should not be kept too moist, and the bath is but ill calculated to restore them. The circulation in those parts being but naturally slow, the bath would increase the evil, nor can they ever be cured by turning out, without shoes.

The shoe mostly used for such feet is generally a broad-heeled shoe, which is made to fit the web, and bevelled away in the inner surface to receive the convexity of the sole, without pressing on it; however, to make the best of a bad bargain, such feet are only fit for the ploughed field.

### OF FIRING:

### AND HOW TO PERFORM THE OPERATION.

The operation of firing is important, and a good and salutary agent, in the hands of those who will use caution and discretion in its performance. The practice of firing is as old as Methuselah, and from time immemorial has been known as not confined to quadrupeds only: on the contrary, among the Africans and many of the Hindoo race, it has been practised for centuries past, and even to this day is generally used among themselves as well as their cattle for the prevention of many diseases; and in many parts of India, on the Ganges, is by the Brahmin physicians applied, instead of our blister, over the abdomen, for the cure of the cake ague, as so called by them, and which is nothing more than a schirrosity of the liver; they also use it for white swellings and numerous complaints; nor would it be difficult to prove, as is observed by a celebrated practitioner of the day, professor Blane, that we have no remedy among us, except mercury, that can compensate for its disuse. Firing is performed on horses for two purposes; one for performing a permanent bandage to a part, which it does by destroying the elasticity of the skin, and the other is to produce an active inflammation, and thereby excite absorption; and it is even common to this day among the Arabs, to fire the joints of their young colts, under the same idea as was some years ago practised on the English turf, to strengthen their limbs; and I have seen them, as well as the Hindoos, perform this operation in various ways all over the carcass of their horned cattle while very young. In Splints, Spavins and Ring-bones, it is used as a strong stimulus to force the surrounding vessels to remove by absorption the substances deposited. The various cases in which firing will be found useful, are particularly detailed in this work; it is, therefore, quite unnecessary to enumerate them here. It is true, I have seen this operation performed on horses without even their flinching, at the same time it need only be remarked, that as it is a painful operation, so it should never be resorted to but when absolutely necessary. When absorption only is required, blisters may be used, as they act in a similar way, which renders the other operation quite unnecessary. I have stated in a former part of this work, that blistering is often necessary immediately after the cautery; but it is only admissible in certain circumstances, and in common cases may be avoided. The practice of the College is, to fire and not repeat the blister till the third, fourth or fifth day, according to circumstances; and the operation of firing in that school is conducted differently in different cases. Mr. Sewell generally fires in straight lines, and they may be perpendicular or horizontal, but should never cross each other: however, do it which way you will, the intention is the same; but the College recommends the lines to be perpendicular, and these I have invariably found to be the best, excepting in cases of breaking down as farrier's call it, or rupture of the suspensory ligament or tendon: in this case I have for many years followed the practice of the Arabs and Hindoos, which is to fire vertically round the affected limb, in which case you had better suspend your horse's legs in the air, or rather truss him up with bundles of straw, with his back under a beam; and in this way you can the more readily work around the affected limb. In firing the pasterns I generally do it in a penniform manner, as represented figs. 3 and 4, plate II; but in Curbs, Splints, and Spavins, I generally carry my lines at angles, avoiding the cross fire if possible. The stifle is usually done the same; but that of the round bone or articulation of the thigh, is generally made to represent a star for neatness. In ring-bones the lines of fire, by one of my worthy instructors, Mr. Bracy Clark, is recommended perpendicularly, and about an inch and a half apart. This is the most handsome firing operation I ever saw for neatness. However, when the principle of the operation is understood, the form and direction of the lines may be left to the fancy of the operator. According to the parts to be fired, so different formed irons are used; but the principal is the searing iron, the budding iron, for cavities in the neck after bleeding and open joints, and the common firing iron, all which should be made sufficiently thick to retain the heat, and should never be heated to a *white*, but a *red heat* only.*

In firing in lines care should be taken that by repeated heating the iron does not form too sharp an edge, or the skin may be fired through, and then you have a permanent blemish. However, to prevent this, after each heating the iron should be rubbed on a smooth board, to round it, and also to loose the *scoriæ* or roughness that may be attached. The best mode of heating your iron, is to have three or four in the fire at a time, and that made of charcoal in a chafing-dish, or a hole in the ground, and that also near the operator. This will save trouble, and expedite the business. I must, however, again particularly caution the operator, that under no consideration to fire through the cuticle; for if the cutis or true skin is wounded, inflammation and ulceration follows; and to prevent this, if your iron is very hot, press it more quickly and more lightly, but as it cools more leisurely and with more pressure. All Ring-bones and Bone Spavins require the severest firing, but

* On this subject of heating the iron, simple as it may appear, I could write pages for the instruction of the smith in this branch of the shoeing trade, if I only thought he would benefit by it, there being no one part of his profession that he should study more than the proper temperature of his iron, for every kind of work; but when you point out these things for his better information, finding there is more labour attached to it, he kicks immediately, and calls you a d——d fool in the bargain.

even in these cases never go through the skin; though I have in some instances seen, and I believe this is professor Blane's (a nice operator) practice, purposely to perforate the skin, yet he does it, I am informed by Mr. Youatt his partner, only in very bad cases, and then as a desperate remedy, which always excites very high inflammation. The best mode of judging of the depth of your fire is the colour, which should be a yellowish brown, not unlike buckskin breeches; and in all cases the hair should be cut close, for without this precaution, the smoke from the hair impedes the sight; and to draw them correctly, Bracy Clark's invariable rule is to have chalk lines, and so pass the fire over them, and when the weather is very hot, and you do not want to blister the part, nothing more is necessary than to rub a little tar on the blistered part, and then apply shag cullings of tow to keep away the flies, after which, in a day or two, you may apply some hog's lard, or other grease to prevent the cracking of the skin.

### OF SELLENDERS AND MALLENDERS.

Mallenders is generally termed a cutaneous disease, and is mostly confined to draught and heavy horses. It is an inflammation of the integument below the hock, which occasions cracks, from which ooze a fetid matter, and is generally produced by an obstruction of the perspirable cutaneous and capillary vessels of the skin, from want of cleanliness, friction, and proper grooms.

There are a variety of modes of cure; but that which I have ever found the best, though not exactly conformable to the College practice, is to clip off the hair, and wash off the scurf with soap and water. Then apply the following ointment:

| | |
|---|---|
| Mercurial ointment . . . . . | 4 drachms. |
| Camphor . . . . . . . . . . | 1½ do. |
| Burnt alum . . . . . . . . . | 2½ do. |
| Honey, Q. S. *Mix.* | |

OR,

| | |
|---|---|
| Alum burnt and pulverized . . | 3½ drachms. |
| Zinc, or white vitriol . . . . . | 5 do. |

Molasses, honey and lard Q. S. to mix into an ointment.

*Sometimes a purgative or a diuretic ball is necessary.*

When this disease of the integuments exhibits a scurfy or scabby eruption at the posterior bend of the knee, it is called Mallenders, and when it appears at the bend of the hock in front, it is then called Sellenders. They neither do harm nor cause blemish, though sometimes troublesome to remove. The College treatment is:—

| | |
|---|---|
| Subacetate of lead, or sugar of lead, or ex saturn, whichever you please to call it . | 1½ drachms. |
| Camphor . . . . . . . . . . . . | 2 do. |
| Ointment, mercurial . . . . . . | 1 ounce. |

*Mix and wash.*

### FRACTURED BONES

### OF THE EXTREMITIES.

On this subject but little can be said, excepting that if the horse be a stallion, and of great value, every mean in the power of man should be devised to set and unite the parts by strings, bandages, &c. For a blood brood mare the same will apply—if not, destroy her.

### OF A STRAIN

### IN THE JOINT OF THE WHIRL, OR ROUND BONE.

A celebrated author of the present day observes, as chest founder covers all other defects of the fore limbs, so are all lamenesses behind referred by farriers of the old school, to either a strain of the round bone or stifle joint, as their fancy leads them to favour the one or the other, and improper treatment may sometimes injure the ligaments of the articulation of the thigh with the pelvis.

*Treatment.*

From the very deep situation of the parts, the cure is generally attended with some difficulty. It will therefore be highly necessary to foment, or apply saturnine lotion till the inflammation is reduced; after which, blister actively. It is not improbable, that the practice of pegging, as formerly practised by the older farriers, might here prove, from the depth of the affection, to be a useful stimulant, if properly applied. But if the parts do not readily reinstate themselves, fire over the joint in a star like form, blister, and when this is healed, apply upon the part a charge of pitch, aloes, and turmeric, each of equal quantities, melted over the fire with strong brandy or spirits of any kind, and reduced to a thick liniment or paste, to which a little rosin or wax may be added to make it stick.

### AN ARRANGEMENT

OF THE

PREVALENT DISEASES

OF THE

### HORSE, AND OTHER QUADRUPEDS:

WITH THE

REMEDIES MOST PROPER FOR THEIR REMOVAL.

### BLISTERING OF HORSES.

Blistering of horses is of great advantage in inflammations, to remove old swellings, strengthen old weaknesses, and to take away Splints, Curbs, Spavins, &c. For all these the blistering ointment will be found excellent.

### BREAKING DOWN.

Breaking down may be much assisted by bathing the parts well with the veterinary opodeldoc, or embrocation for strains; and when the heat is removed, apply the blistering ointment, or bath, with liquid blister.

### OF BROKEN WIND.

Cough balls and blistering the wind-pipe, with regular regimen, plenty of carrots, potatoes, beets, &c. will sometimes, in the early stages of the complaint, remove it; but when confirmed, and the air cells of the lungs become ruptured, there is no remedy.

### OF COLDS.

When a horse has caught a cold, the fever powders will remove it; but should great weakness and debility remain, give the cordial fever ball.

### CONDITION.

When horses are out of condition, warmth, and the alterative condition powders, will restore it.

### CORDIALS.

When cordials are necessary, give one of the cordial balls.

### COUGH.

When a horse has a cough without fever, give a course of cough balls and he will speedily get relief.

### OF CURBS.

A Curb is generally removed by one or two blisters of the blistering ointment, though they sometimes remove themselves.

### DIURETICS.

Diuretics are excellent cleansers; they not only promote condition, but remove swelled legs, and are also good in strangury or obstructed urine or gravel.

### EXERCISE.

Exercise is of all things essentially necessary to horses, but when from lameness or bad weather they cannot go out, they should be well hand rubbed, and one of the diuretic balls given each other or third day, which will keep their legs fine.

### OF THE EYES.

When the eyes are swelled from inflammation, relief may sometimes be obtained from the eye water; but when from blows or bruises, sometimes hot and sometimes cold fomentations will remove it.

### OF FISTULA SORES.

All fistula sores, or pole evil, those of the withers, &c. may be removed, if not very inveterate, by injecting into them either the liquid sweating blister, or mild wash for Grease; but when they have become very inveterate and foul, the strong paste for Grease must be melted and scoured in.

### OF CLYSTERS.

Clysters are under almost all circumstances useful to horses. Various forms of them will be described.

### OF GREASE.

Grease, in its early stage, may be cured by the mild wash or astringent ointment; but when become inveterate, the strong paste for Grease is necessary, with other diuretic medicines.

### GRIPES OR COLIC.

Gripes or colic may be removed by clysters, raking, and the proper diuretic ball.

### HIDE-BOUND.

Horses hide-bound should be treated with a course of alterative balls and condition powders, which with good grooming will generally remove the complaint.

### JAUNDICE OR YELLOWS.

This is a liver complaint, and may always be removed by a few doses of mercurial physic.

### LAMENESS.

Lameness, when arising from strains, may always be removed by the application of the veterinary opodeldoc, or embrocation for strains, or the liquid blister; when it arises from Splints, Bone Spavin, Blood or Bog Spavin, Ring-bones, or other causes, the latter will be found to be the best, but partiticularly where firing is necessary.

### MALLENDERS OR SELLENDERS.

Mallenders or Sellenders can always be removed by the Grease paste or ointment.

### MOULTING.

Moulting, in the spring of the year, has very often on the constitution of some horses a very severe effect, in rendering them faint, weak, and incapable of going through their daily labour. The alterative balls given every night and the cordial balls every morning will remove them.

### OF POULTICES.

The beneficial effects of these, under their various forms, will be found very useful, as described under their several heads.

### PHYSICKING OF HORSES.

Physicking of horses, although sometimes necessary and useful, yet if constantly practised in the spring of the year, it frequently proves injurious. For swelled legs, for grease, hide-bound, &c. the balls will be found very useful; they are of various sizes and strength, as enumerated in this treatise.

### QUITTOR.

Quittor may be cured by the knife, and the introduction of some of the Grease paste or other applications, as enumerated in the work.

### RING-BONES.

Ring-bones may be removed by firing and the blistering ointment.

### SPAVINS AND SPLINTS.

These may also be removed by the same applications.

### OF STABLES.

The economy and ventilation of stables will be largely treated of in the body of the treatise.

### STRANGLES, CATARRH, AND INFLUENZA.

These are nearly synonymous, and will also be treated of very largely in the body of the work.

### SURFEITS.

Surfeit may always be removed by a course of alterative and condition powders.

### THOROUGHPINS.

Thoroughpins may be removed in the same way as Splints and Spavins.

### RUNNING THRUSH.

Running Thrush you will find treated of very largely in the work.

### WIND-GALLS.

Wind-galls may be removed by a little blistering ointment, the embrocation for strains, and bandage, &c. For their total removal, you must fire; but they are most generally an eye-sore, and should never be touched.

### OF WORMS.

Worms are very troublesome to horses, and sometimes dangerous; they may be removed by mercurial physic, and the worm powder sprinkled on the food.

### THE MEDICINES

FOR

### The Ox, the Cow, the Sheep, and the Dog,

Will be treated of in the work, and the proper quantities specified. The doses must not be so large as for the horse; treatment nearly the same.

---

### *FOR THE DOG.*

A course of alteratives is very useful for dogs, in removing the mange, killing worms, and promoting their condition as well as cleaning their coats.

### CANKER IN THE EARS OF DOGS.

This disease may be easily cured, by pouring on the wash for canker in the ear.

### CONDITION OF DOGS.

When dogs are out of condition from mange, foul feeding, foul coats, worms, &c. they may be brought round by the mange condition powder or bolus.

### DISTEMPER IN DOGS.

Distemper is a term very much confounded and misunderstood in dogs as well as horses. The distemper powders will remove it. But this complaint, being a very interesting one, will be treated of more largely.

### MANGE IN DOGS.

Mange in dogs, in all its varieties, may be cured by the application of the mange ointment.

### PHYSIC.

The best physic in the world for a dog is castor oil, or the condition physic.

### WORMS IN DOGS.

Worms may be removed by the worm powder.

### ON CASTING HORSES;

### WITH A DESCRIPTION OF THE NEW HOBBLES,

*By Bracy Clark, F. L. S.*

THIS improvement in the apparatus for casting down horses, and better securing them for operations,* presented itself to me about six years ago; the great advantages of this plan having been fully confirmed by frequent use during this period, I have been induced to make it more publicly known.

In casting horses after the common mode, some straw, enclosed in a knot, was used to form an obstacle to the return of the rope through the four rings, and, in order to insert it, it became necessary to relax the rope for a time, during which, if the horse struggled, he would draw the cord back through the rings, and sometimes get upon his legs again, a circumstance we have seen to happen; this imperfection is

* Although we have treated largely on the subject of firing in the body of the work, it is necessary to remark here, that fig. 1, in the plate, represents the direction of cross lines, and although they sometimes are preferred, I myself do not approve of them, and my reason is this, I seldom or ever saw lines drawn in this *cross direction*, but if the iron happened by accident to go too deep, that the square did not sluff and leave a permanent blemish. To fig. 2, this is also applicable.

In firing for a Curb or Spavin, I should recommend the iron to be drawn in lines like fig. 3, on each of the lower and posterior parts of the hock. For Wind-galls I would also do the same. The straight lines, as represented in fig. 5, are very good. When a horse is to be fired in both legs, it matters not on which side you cast him. As respects circular rings round the leg for Wind-galls on the pastern, it is a method uniformly practised by the natives of the East, of which I have witnessed the best effects. Remember, that when you do this, your rings should never exceed an inch, and if a little closer the better.

A
B
C
D
E
K
1
2
3
4
5
Fig. 2.

wholly removed by the present apparatus. The knot of straw also drawn tighter, and condensing by the struggling of the horse, would give a degree of liberty to the legs that was often inconvenient in performing an operation, which is also removed by the present apparatus, which can be drawn as tight as you please, and afterwards is subject to no relaxation. But a much greater advantage than the foregoing, is, that all the four legs are released at once, and the inconvenience and risk of undoing a leg at a time, after the old mode, is entirely done away. The friction and strain of these new hobbles is chiefly confined to the iron parts, which gives them a duration in wearing that is almost endless. The iron chain terminating the rope, is found to pass or slide through the rings with less friction, if well made, than a rope would do; and, by the use of the steel rollers, it requires much less force, or fewer men in casting the horse.

The apparatus itself is represented in the plate annexed, and the following brief description will enable the reader to form a correct idea of the nature of it:—One of the hobbles, it will be observed, is made different to the rest, which we call the principal or master hobble; it is seen on the near fore leg, and is always to be placed or fixed on the fore leg of that side of the horse that is designed to be uppermost when on the ground; the end of a chain is fastened to this hobble by means of the moveable pin (A), which is prevented from falling out with any agitation of the apparatus, by having two or three turns of a screw in its upper part, under the thumb piece (A). The chain (B) terminates in a swivel, received into the notch through which the pin passes, and the links of this end of the chain are made open and plain, for receiving the hook for fastening him; the rest of the links are twisted and made shorter to run more readily upon the rings; to this chain is attached a cord (C); we have preferred one of those which are called the patent ropes, being plaited of strands of equal tension, and are found not only stronger but more flexible than the common ones. At the extremity of this cord is attached, for convenient use and to prevent its being lost, the oblong curved hook of iron (D).

It would appear almost needless to describe the manner of using or applying the apparatus, as it is so simple, and the plate so plainly indicates it; it is evident, that on drawing the rope, the legs are forced together, and he is made to fall, some straw being placed on the ground to receive him: he is no sooner down and the feet drawn together, than the hook (D) is passed through one of the open links of the chain, which, as it cannot then return back through the perforation in the plate (E), he becomes perfectly fixed, and nothing further is necessary; and far more conveniently and effectually, than by any noose or knot that could be devised.

The operation being performed, the removal of the pin (A) sets him at liberty again, as the extremity of the chain can then pass without impediment through all the rings.

We may observe that each hobble ring, except the *principal*, is provided with a steel roller or case of plate-steel, which, turning readily upon the iron, serves to diminish the friction, though it has been found, if made simply, of a large half-round iron ring, in which way this apparatus has been several years used in the king's body guard, to answer particularly well, yet we think the rollers preferable; and it may not be without its use to remark, that if the steel moveable cases or rollers are resorted to, they should be made of very stout plate-steel, so strong that the chain should not, in passing through the rings, or in the struggles of the horse after he is down, be subject at all to force or indent them, which would render them perfectly useless.*

Some persons have expressed a desire, that not only the

* Dr. Carver has appointed Mr. Dixon, saddler, in Market street between Seventh and Eighth streets, his agent for making these hobbles, Dr. C. having supplied him with a complete model for that purpose.

legs should be set at liberty when the chain leaves the hobbles, but that the straps also should disengage themselves from the legs; a proposition that appeared truly difficult; nor will this circumstance, in actual practice, be found of so very great moment as might be imagined; however, this difficulty we have overcome, and the proposition is fully accomplished, by the use of the hobble, fig. 2, having no buckle at all, but is furnished with two rings, attached to the two ends of the strap, which, with the ends of the strap, fly open as soon as the chain is drawn away and falls from the legs. The little chain and hook (K) serves to hold the two ends or rings together about the leg, during the introduction and passing of the chain; there is provided also to these rings, moveable rollers, both to the upper as well as front parts, which are found greatly to assist in destroying the friction, the chain having, during the draught, a strong pressure upwards.

As casting the horse adroitly will greatly recommend the veterinary practitioner in the eyes of a discerning spectator, so we shall subjoin a few hints and cautions which practice has taught us for effecting it more easily and with less risk of accidents or injuries.

With high-spirited, shy, or skittish horses, the four hobbles cannot be applied with too much expedition; there should be a man to each leg, and the rope previously passed through the rings, and made fast to the pin, with men ready at the rope in case of any sudden movement or struggling on finding himself hampered, to pull him down immediately, and prevent his moving from the spot; and, on all occasions, the horse should be blinded previously to the hobbles being applied, and a twitch to the nose or ear is also useful to divert his attention. The blinding-hood should be of a very coarse, thick cloth, and made to cover the head and upper part of the neck, with ties below of ferret or tape; it is best applied by being laid upon the crest and gradually advanced forwards over the head and eyes, and then tied, which is always to be undone, but not removed till he is fully at liberty for getting up. The smith's apron usually was resorted to in these cases for blinding; it is but a very uncertain and indifferent blinding-hood. This hood cannot be made of too thick materials, as it may be very useful also to defend the head and eyes from injury after the fall, and during the operation.

In the plate annexed, the legs of the horse are seen approaching each other out of the perpendicular, and drawn towards the centre of the body, by which the power of resistance is greatly diminished, and into which position they may mostly be brought before the men begin to pull the rope, or the horse is aware of being hampered or disposed to make any resistance; and if only one or two of the legs are so situated, it will have its effect in facilitating the fall without his moving from the place on which he stands: but, with some very shy horses, it is best not to make any attempt of this sort; with heavy horses or draft horses, &c. it is more particularly of advantage. But when it is practicable, it is most advantageous to get the two legs of the off or opposite side to the men pulling at the rope into this situation, being the side on which he is to fall; as he then more certainly falls to that side. Some horses spring from the ground, when they find themselves entangled, in which case their fall becomes more uncertain; if, by chance, they fall towards the rope, they must be turned over on their backs after the hobbles have been made fast by the hook.

The horse's tail, as having great command of him under the circumstances of his being poized and about to fall, has been usually my post of duty in this process, and frequently, in the moment of balancing, the opportunity is afforded of deciding the fall on the side we wish; a steady confidential man also should be placed at the head, and another of this description should manage the rope next the hobbles, taking a short hold almost close to the apparatus, pushing with the other hand against the shoulder or side of the horse, and renewing his hold as the rope passes; this, however, requires some address, as the horse may, if it is resorted to too soon, lean towards it, which they are apt to do when any thing is pressing against them, and so fall on the near side, that it should be desisted from if this is likely to take place, but may much assist if duly used.

In the moment of pulling the rope stationed at the tail, I take the opportunity with my foot of touching pretty sharply the hind leg on the off side, or side from the casting rope, and, as he lifts up, it is drawn inward towards the centre of the body, and greatly assists in the operation, determining also in general his fall to that side.

One hint more I might also subjoin, in respect to handling the horse, for it is very common to see the shoeing smiths about any operation of this sort of a painful kind, approach the horse with a timidity of suspicion, and touch his legs and other parts with their fingers' ends, thereby creating an unnecessary alarm and irritation: he should, on these occasions be approached with confidence, and the flat hand be used with the greatest freedom.

There is a small part or addition to this apparatus, which I have not yet described, which is a loose hobble, or noose, for taking out a leg, if necessary, or to tie it in any position to the other legs, that may be required for the conveniently performing an operation, or, in case of accident, it may be used to secure him: if made of a rope with a flat webbing at the end, and an iron ring to pass the rope through, it answers very well, for such we have used; and, it may be almost superfluous to say that two or three trusses of straw should be scattered on the ground to throw him upon.

In this way, the most painful operations may be performed upon this powerful animal with perfect safety; and I rejoice the more at the success of these means, as I well remember, in the commencement of my studies in this art, that casting the horse was attended with so much trouble and difficulty from being badly performed, that instead of it, severe twitches were had recourse to, during an operation, applied to the nose as well as ears; and he was often cruelly beaten with whips and sticks, to make him stand up during the performance of firing or other operations, to make him stand quiet, and was often in this manner most shamefully abused, which now there can be no just cause for resorting to.

As it is by practical improvements, the veterinary art must be advanced in utility and estimation with the public; so I shall feel happy, if this little invention may be considered in a small degree contributing thereunto.

## Figures and Tables

**Fig. 2. f1:**